# Cardiovascular Anthropometry: What Is Best Suited for Large-Scale Population Screening in Sub-Saharan Africa?

**DOI:** 10.3389/fcvm.2020.522123

**Published:** 2020-12-03

**Authors:** Hadiza A. Agbo, Ayuba I. Zoakah, Christian O. Isichei, Atiene S. Sagay, Chad J. Achenbach, Basil N. Okeahialam

**Affiliations:** ^1^Department of Community Medicine, Jos University Teaching Hospital, Jos, Nigeria; ^2^Department of Chemical Pathology, Jos University Teaching Hospital, Jos, Nigeria; ^3^Department of Obstetrics and Gynaecology, Jos University Teaching Hospital, Jos, Nigeria; ^4^Department of Medicine, North Western University Feinberg School of Medicine, Chicago, IL, United States; ^5^Department of Medicine, Jos University Teaching Hospital, Jos, Nigeria

**Keywords:** anthropmetry, abdominal height, body mass index, waist to hip ratio, prediction, cardiovascular disease

## Abstract

**Background :** Body mass index (BMI) measures overweight/obesity. It, however, especially in sub-Saharan Africa (SSA), misclassifies cardiometabolic risk. Central obesity measures are superior. We therefore sought to compare BMI, waist-to-hip ratio (WHR) and abdominal height (AH) in predicting cardiovascular disease risk in sub-Saharan Africa.

**Methods :** Subjects had blood pressures, BMI, and WHR determined. Blood pressure was taken, weight and height measured to generate BMI, and AH measured with a new locally fabricated abdominometer. The ability of the anthropometric indices in identifying abnormal individuals needing intervention was assessed with sensitivity, specificity, and area under the receiver operator characteristic curve.

**Results :** Adults totaling 1,508 (728 M/780 F) adults were studied. For BMI, 985 (65.3%) were normal, while 375 (24.9%), consisting of 233 males and 142 females, had normal WHR. Blood pressure was normal in 525 (34.8%) and 317 (21.0%) for systolic and diastolic blood pressures, respectively. Using BMI as gold standard, sensitivity, specificity, positive, and negative predictive values for WHR in males were 80.7, 37.5, 62.5, and 19.3%, respectively. For females and in the same order, they were 62.0, 34.3, 65.7, and 38.0%. For AH, it was equal in both genders at 82.6, 39.2, 60.8, and 17.4%. By receiver operating curves comparing AH, WHR, and BMI against blood pressure detection, the area under the curve was 0.745, 0.604, and 0.554 for AH, BMI, and WHR, respectively.

**Conclusion :** Abdominometer-derived AH has a better sensitivity and greater area under the receiver operator curve compared with BMI and WHR in this sub-Sahara African population; implying superiority as a cardiovascular anthropometric index.

## Introduction

Obesity and overweight are associated with development of cardiovascular diseases (CVD) such as hypertension, diabetes mellitus (DM), and the metabolic syndrome, a conglomeration of CVD risk factors (1). According to the World Health Organization (WHO), obesity, and overweight refer to abnormal or excessive fat accumulating in the body, which in turn impact negatively on health (2). This realization that overweight and obesity have an adverse effect on health has been recognized as far back as the 6th century BC (1). The WHO therefore came up with the body mass index (BMI) as measure of overweight and obesity for use in epidemiological studies (3).

It has been known that, whereas the BMI increases with body weight, the weight increase may be due to different reasons spanning from increase in muscle mass, adiposity, or bone density. This makes (BMI) a poor risk discriminator as it does not distinguish between weight increase from fat, lean muscle, or bone (4). This derives from the fact that all pathology arising from overweight and obesity is due to excess fat mass (5). Therefore, attention shifted to percentage body fat as a better anthropometric index for CVD prediction (6). Apart from bioelectrical impedance analysis, other methods of assessing body fat are cumbersome and do not yield themselves easily for large epidemiological studies (7). Notwithstanding this limitation, the fact that it is not just fat but its location in the body that relates to cardiometabolic disease (8) further shifted attention to measures of central obesity. This followed the finding that visceral adipose tissue is the chief contributor to cardiometabolic diseases (9, 10). The result was development of anthropometric indices like waist circumference (WC), waist-to-hip ratio (WHR), and waist-to-height ratio (WHtR) ratio, which proved to be better than BMI (11, 12). BMI was therefore shown to miss subjects with cardiometabolic risk factors related to increased adiposity (13).

Coming home to sub-Saharan Africa (SSA), this WHO standard has shown to be inappropriate (13), as genetic factors modify its association with CVD risk (14); calling for the need to find some other appropriate anthropometric measure. W, which is simple and works well among Asian populations, does not always reflect visceral obesity that includes abdominal subcutaneous fat (9), which is a metabolic sink. It also does not factor in individual and ethnic differences in phenotype and body build (15). Writing on the subject, Lin et al. (16) posited that anthropometric measures in different ethnic groups have different predictive powers in cardiometabolic diseases, hence, the need to establish for each index appropriate cutoff points. This gave rise to the abdominometer concept with the abdominal height (AH) being the anthropometric measure considered appropriate for SSA (17). This differs from the South American version, which measures sagittal abdominal diameter (18). In this case, a caliper is fixed on the bed, and it measures the abdominal height. By lying down, part of the abdominal fat is bound to be displaced laterally and, hence, missed in the measurement. It is also not amenable to use in the field for epidemiological studies given its bulk. Our version is a light portable piece of furniture used with the subject standing erect and can be taken far afield for epidemiological studies. It has been tried in restricted populations with good results (19, 20).

This is therefore an attempt to use the abdominometer as conceptualized by Okeahialam (17) to study a large population of free living adults in North-Central Nigeria and compare the suitability of AH for large epidemiological studies as opposed to BMI (the WHO standard) and WHR, which measures central adiposity. If found appropriate, given the ease of use, it could become a tool to screen populations for CVD especially in SSA; since the WHO standard, the BMI has failed in many instances to attribute CVD risk to individuals.

## Methods

### Study Area

We purposively selected Mangu Local Government Area in the central part of Plateau State, North-Central Nigeria for the study given our experience with the locality in previous studies (21, 22). The area is home to persons of diverse ethnic affiliation though predominantly consisting of the Mwaghavul, Pyem, and Fulani.

### Study Population

This was made up of adults 18 years and above, males, and non-pregnant females who consented and gave written approval to participate in the study in accordance with the Helsinki Declaration. The Research and Ethics Committee of Jos University Teaching Hospital gave approval.

### Design and Sampling

This was a descriptive, comparative cross-sectional study. By simple random sampling using balloting, two wards, Gindiri 1 and Langai, were selected from which six communities were further selected randomly. They were Kasuwan Ali, Angwan Bature, and Nagwak from the former, while Langai, Buli Rumada, and Buli Kedung were from the latter. An advocacy visit was earlier undertaken to inform all households of the designated site and date of data collection. Participants were documented on arrival in the research register, which formed the sampling frame.

### Tools/Techniques of Data Collection

An interviewer-administered semistructured questionnaire was used to collect sociodemographic data. A weighing scale (Hana Model) was used to measure the weight of the participants in kilograms. The scale was reset to zero every day before use. Subjects were minimally clothed for this and stood erect. A stadiometer calibrated in meters was used to measure height. From these measurements, BMI was arrived at using the formula w/h^2^. Blood pressure was measured using a calibrated digital apparatus [Omron (R) M2 Basic] on two occasions. Subjects were seated for at least 5 min before the first measurement, and a repeat was done after at least 2 min. The average was taken for assessment. A flexible measuring tape was used to measure WC by applying it midway between the lower rib and iliac crest in expiration, and the result was documented in centimeters. For hip circumference, the tape was applied at the level of the trochanters and the outermost protrusion of the buttocks, and the reading was taken in centimeters. From these, the WHR was derived.

**Figure 1:**
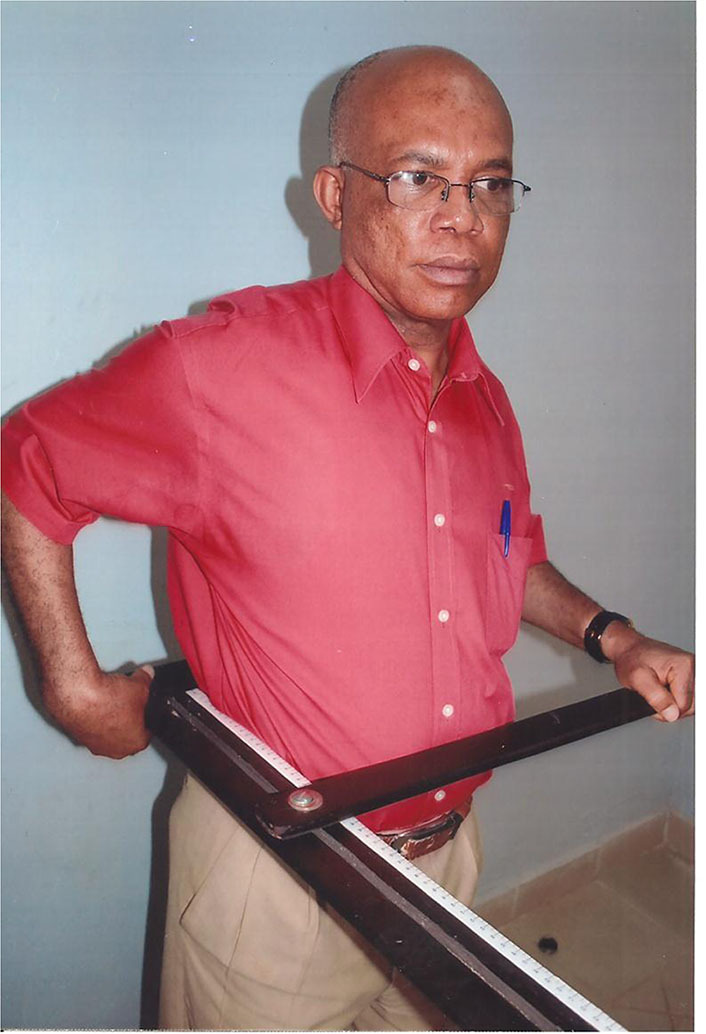
Abdominal height measurement. The corresponding author (BO) using the abdominometer, which he conceptualized by himself to demonstrate its use. He unreservedly gives informed consent for the use of this image for publication. The short arm is sitting in the small of the back at L4/L5 interspace. The long arm is at the side, and the swinging arm is brought forward to make contact with the abdomen at the level of the umbilicus and height read off on the graduation.

The AH was measured with the abdominometer, a light wooden apparatus conceptualized by Okeahialam (see Figure 1) and used in earlier pilot studies (17, 19). It is applied with the subject standing erect with the short arm of the “L” -shaped apparatus sitting in the small of the back at the level of the posterior iliac crest, at lumbar 4/5 intervertebral space. The sliding arm is then activated with the swinging arm brought to rest anteriorly at the anterior-most part of the abdomen. The distance is now read of in centimeters on the sliding scale strapped to the longer arm.

BMI was considered normal if it was between 18.5 and 24.9 kg/m^2^, overweight if it was between 25 and 29.9 kg/m^2^, and obese if 30 kg/m^2^ or above. WHR was considered normal if it was ≤ 0.89 and 0.79 for males and females, respectively (23). For AH, <22 cm and <21 cm were considered as normal for males and females, respectively (24). The reliability and efficacy of the different cardiovascular anthropometric indices were then compared.

### Data Analysis

Microsoft Excel was used for data entry and cleaning, while statistical analysis was performed with SPSS Version 20.0 software package (SPSS Inc. Chicago IL, United States). Quantitative and qualitative parameters were obtained. A frequency table was generated from the sociodemographic characteristics (sex, age, marital status, parity, and religion). The predictive ability of the anthropometric indices in identifying individuals, normal or abnormal, and hence in need of some intervention was assessed with sensitivity, specificity, and area under the receiver operator characteristic (ROC) curve. Statistical significance was set at a *p* < 0.05.

## Results

A total of 1,508 free living adults were studied, 728 of whom were males and 780 females. Islam was the predominant religion. The detailed sociodemographic characteristics are shown in Table 1.

**Table 1:** Sociodemographic characteristics of the study population.

**Characteristics**	**Frequency (*n* = 1,508)**	**Percentage (%)**
**Age groups(years)**			
18–27	381	25.3
28–37	389	25.8
38–47	241	16.0
48–57	199	13.2
≥58	298	19.8
**Sex**			
Male	728	48.3
Female	780	51.7
**Marital status**			
Single	376	24.9
Married	985	65.3
Separated	5	0.3
Divorced	15	1.0
Widowed	127	8.4
**Religion**			
Christianity	498	33.0
Islam	1,007	66.8
**Parity**			
<4	729	48.3
≥4	779	51.7

Using cutoff values earlier mentioned, 985 (65.3%) had normal BMI, while 375 (24.9%) made up of 233 males and 142 females had normal WHR. Blood pressure was normal in 525 (34.8%) and 317 (21.0%) for systolic and diastolic blood pressures, respectively. The median values of the systolic and diastolic blood pressures were 129.00 mmHg (range: 98–170) and 79.00 mmHg (range: 65–100), respectively.

By cross-tabulation of BMI vs. WHR and AH in order to determine sensitivity, specificity, positive, and negative predictive values, a table was generated (see Table 2). Using BMI as gold standard, and deriving from Table 2, the following results were obtained.

- WHR male: Sensitivity–80.7%; Specificity–37.5%; Positive predictive value (PPV)–62.5%; Negative predictive value (NPV)–19.3%.
- WHR female: Sensitivity–62.0%; Specificity–34.3%; Positive predictive value–65.7%; Negative predictive value–38.0%.
- AH male: Sensitivity–82.6%; Specificity-39.2%; Positive predictive value–60.8%; Negative predictive value-17.4%.
- AH female: Sensitivity–82.6%; Specificity–39.2%; Positive predictive value–60.8%; Negative predictive value–17.4%.

**Table 2:** Cross tabulations of gold standard test [body mass index (BMI)] and screening tools.

**Screening tools**	**BMI**		
	**Normal**	**Abnormal**	**Total**
**Waist-to-hip ratio (WHR)**			
**Male**			
Normal	188	45	233
Abnormal	797	478	1,275
**Female**			
Normal	88	54	142
Abnormal	897	469	1,366
**Abdominal height**			
**Male**			
Normal	257	54	311
Abnormal	728	469	1,197
**Female**			
Normal	68	33	101
Abnormal	917	490	1,407
Total	985	523	1,508

**Figure 2:**
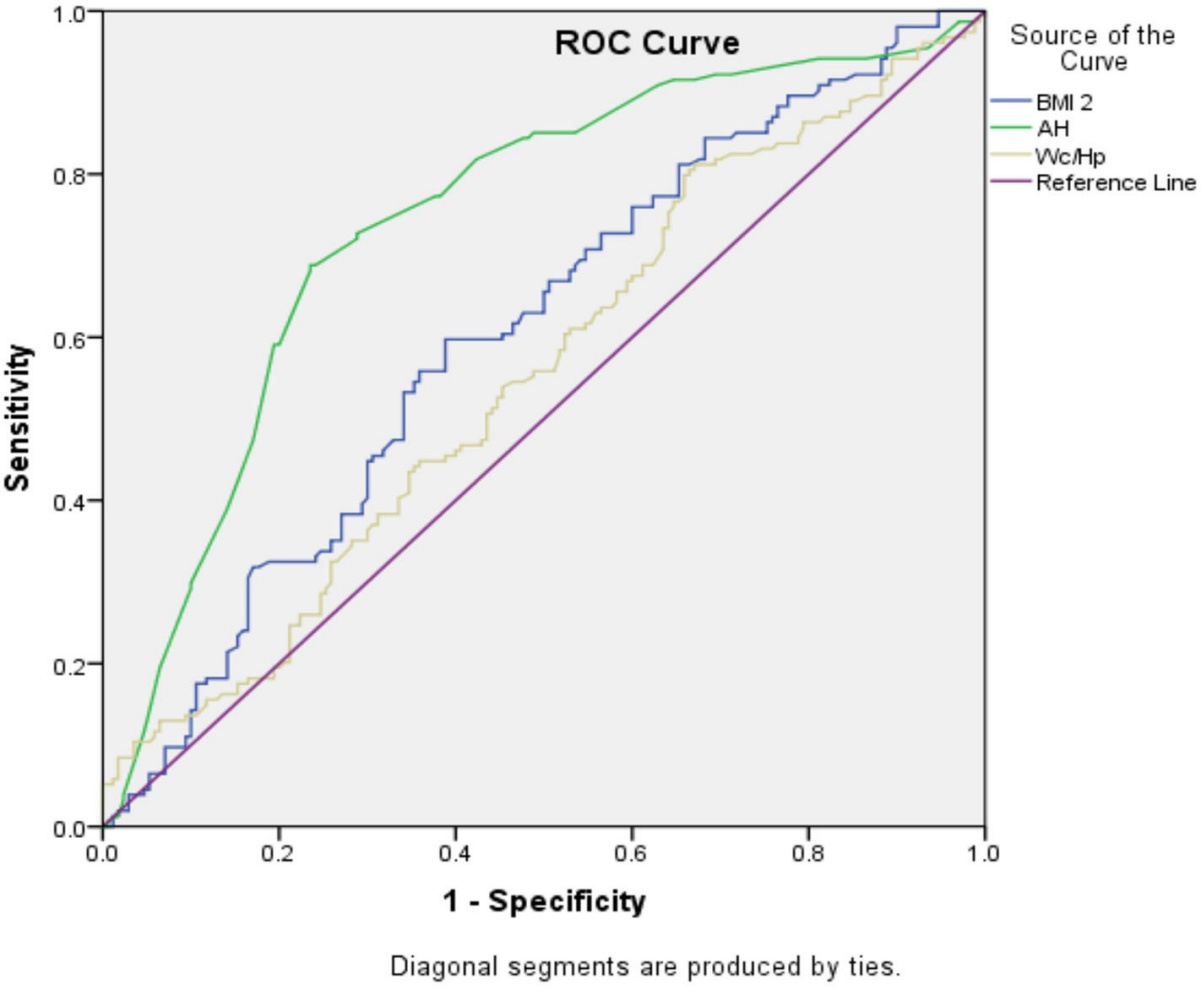
The receiver operating curve (ROC) comparing body mass index (BMI), abdominal height (AH), and wasit-to-hip ratio(WHR) with outcome of abnormal blood pressure.

Receiver operator characteristics generated in comparing sensitivity and specificity of AH, WHR, and BMI against detection of hypertension are shown in Table 3. The area under the curve was highest for AH. Translated graphically in Figure 2, the area under the ROC curve was evidently highest for AH as well. When sex was factored in, AH still outperformed the other anthropometric indices (see Table 4). Subjecting the data to multiple logistic regression, only AH registered a significant value, accounted more for by females. The adjusted odds ratio with 95% confidence interval and *p*values are as follows: AH for males (0.96; 0.735–1.749; 0.753), AH for females (1.91; 1.225–2.971; 0.004), WHR for males (1.12; 0.838–1.507; 0.434), and WHR for females (1.01; 0.707–1.441; 0.959).

**Table 3:** ROC comparison of abdominal height (AH), WHR, and BMI against BP detection.

**Test result variable(s)**	**Area**	**Std. error^**a**^**	**Asymptotic Sig^**b**^**	**Asymptotic 95% confidence interval**	
				**Lower bound**	**Upper bound**
**AREA UNDER THE CURVE**					
BMI 2	0.530	0.017	0.083	0.498	0.563
AH	0.554	0.017	0.002	0.520	0.588
Wc/Hp	0.530	0.018	0.091	0.495	0.564

**Table 4:** ROC comparison of AH, WHR, and BMI against BP detection with sex.

**Test result variable(s)**	**Area**	**Std. error^**a**^**	**Asymptotic sig^**b**^**	**Asymptotic 95% confidence interval**	
				**Lower bound**	**Upper bound**
**AREA UNDER THE CURVE**					
BMI 2	0.604	0.031	0.001	0.543	0.665
AH	0.745	0.028	0.000	0.690	0.800
Wc/Hp	0.554	0.032	0.092	0.492	0.617

## Discussion

AH in this study of a large population of free living adults in North Central Nigeria proved to be the best cardiovascular anthropometric index to predict CVD in our environment. This supports the findings in the earlier pilot studies suggesting that it outperformed the other common cardiovascular anthropometric indices (17, 19) and aligns with studies from other climes (25). This should not come as a surprise. CVD especially hypertension relates more to body fat than total mass (26), but more for fat in the abdominal cavity related to internal organs (27) than sub-cutaneous fat. This is because adipocytes related to viscera generally, but more in the abdominal cavity are dysfunctional and metabolically active (28). In the subcutaneous regions, they are inert and serve as a metabolic sink. BMI has long been used as a measure of obesity, which is known to be a harbinger of cardiometabolic diseases. It later became obvious that it misclassifies risk in individuals. This is because BMI is based on overall weight of the individual, not fat, which is the main risk. Attention then shifted to measures of central adiposity as the basis for classification of cardiometabolic disease risk. This gave rise to other anthropometric measures. With the further finding that the main risk is fat in ectopic sites, attention shifted to fat accumulating in the midsection. Thus, came anthropometric measures like WC and sagittal abdominal diameter as more appropriate in classifying individuals for cardiometabolic disease risk. When in the abdominal cavity or other ectopic sites, fat induces metabolic dysregulation. The consequence is activation of the renin–angiotensin–aldosterone system and sympathetic nervous system, notwithstanding that the individuals are volume expanded. This impairs glucose disposal as well as development of hypertension (29) and contributes to CVD risk (11) by further utilizing indirect mechanisms of atherogenic secretion pattern (30). It could therefore be seen why BMI that developed from mortality data of Euro-American populations would not be appropriate for all other populations including areas in SSA (31). It is from this realization that WHO came to the conclusion that where possible, measures of abdominal obesity should be used to refine BMI in assessment and prediction of CVD risk (32). Abdominal fat being the depot that is most active metabolically has become acceptable as the most appropriate to measure when it comes to assessing for cardiometabolic disease risk. WC is a good measure, but it includes abdominal subcutaneous fat, which is metabolically inert. Sagittal abdominal diameter is equally good, but its measurement requires the individual lying down. Part of the intra-abdominal fat would then disperse to the flanks and become unavailable for measurement when the individual lies supine. Moreover, the equipment used for sagittal abdominal diameter is fixed on a couch, making its use in field epidemiological studies cumbersome.

## Strenghts and Limitations of the Study

The strength of our study is in the sample size and the use of free living adults of mixed ethnicity. It also captures a cross section of socio-economic groups and does not exclude any age range, sex, or religious group. It would, however, for improvement of external validity, be necessary to have included urban residents and extended the study to other geographical locations in Nigeria and, where possible, other parts of SSA. The appliance used here, called the abdominometer, is light, mobile, and amenable for field study use. Its advantage is in measuring the anterior protrusion of the abdomen, which is largely due to the intra-abdominal fat, and with the individual erect, no part of the fat shifts to the flanks. Comparing its ability to predict hypertension among other common anthropometric indices shows it to be a better predictor. It is, therefore, a novel contribution to cardiovascular anthropometry in sub-Saharan Africa where BMI has been shown to be inappropriate. The mobility of the appliance also lends it to easy use in field epidemiological studies, where size, need for electricity, and immobility encumbers other equipment.

## Conclusion

For us in SSA, given the superiority of AH over BMI and WHR, it would appear to be the preferred anthropometric index for predicting CVD risk and triggering necessary preventive or curative action. The mobility and relative ease of use of the abdominometer conceptualized by Okeahialam is an attraction. It is therefore recommended that the abdominometer-derived AH be more widely used for clinical and epidemiological purposes in SSA.

## Data Availability Statement

The datasets generated for this study are available on request to the corresponding author.

## Ethics Statement

The studies involving human participants were reviewed and approved by Research and Ethics Committee, Jos University Teaching Hospital, Jos Nigeria. The patients/participants provided their written informed consent to participate in this study. Written informed consent was obtained from the individual(s) for the publication of any potentially identifiable images or data included in this article.

## Author Contributions

HA was the mentee of the project, collected the data in the field, and performed statistical analysis. AZ is the epidemiologist and supervised the statistical analysis. CI was a co-investigator in-charge of the laboratory analysis. AS was the principal investigator of the project. CA was the oversea mentor of the project and contributed to the design of the study. BO was a co-investigator and the local mentor of project, and wrote the manuscript.

## Conflict of Interest

The authors declare that the research was conducted in the absence of any commercial or financial relationships that could be construed as a potential conflict of interest.
